# A Multi-Laboratory Evaluation of Commercial Monkeypox Virus Molecular Tests

**DOI:** 10.1128/spectrum.00225-23

**Published:** 2023-05-04

**Authors:** Oran Erster, Itzchak Levy, Areej Kabat, Batya Mannasse, Virginia Levy, Hadar Assraf, Roberto Azar, Haim Ben-Zvi, Ritta Bradenstein, Olga Bunder, Ayman Fadeela, Ayelet Keren-Naus, Avi Peretz, Diana Roif-Kaminsky, Lolu Saleh, Licita Schreiber, Orna Schwartz, Pninit Shaked-Mishan, Nadav Sorek, Merav Strauss, Rachel Steinberg, Orit Treygerman, Simona Zisman-Rozen, Ruth Yishai, Noa Tejman-Yarden, Ella Mendelson, Danit Sofer

**Affiliations:** a Central Virology Laboratory, Public Health Services, Ministry of Health, Chaim Sheba Medical Center, Ramat Gan, Israel; b Infectious Diseases Unit, Chaim Sheba Medical Center, Ramat Gan, Israel; c Beilinson-Rabin Medical Center, Petach Tikva, Israel; d Kaplan Medical Center, Rehovot, Israel; e Shamir Medical Center, Beer Yaacov Zerifin, Israel; f Meir Medical Center, Kfar Sava, Israel; g Soroka Medical Center, Be'er Sheva, Israel; h The Baruch Padeh Medical Center, Poriya, Tiberias, Israel; i The Azrieli Faculty of Medicine, Bar-Ilan University, Safed, Israel; j Barzilai Medical Center, Ashkelon, Israel; k Maccabi HealthCare Central Laboratory, Rehovot, Israel; l Wolfson Medical Center, Holon, Israel; m Carmel Medical Center, Haifa, Israel; n Assuta Ashdod University Hospital, Ashdod, Israel; o Emek Medical Center, Afula, Israel; p Me’uchedet HealthCare Central Laboratory, Lod, Israel; q Galilee Medical Center, Nahariya, Israel; r Department of Laboratories, Public Health Services, Ministry of Health, Jerusalem, Israel; s School of Public Health, Sackler Faculty of Medicine, Tel-Aviv University, Tel-Aviv, Israel; Emory University School of Medicine

**Keywords:** monkeypox, real-time PCR, detection kit, comparative analysis, molecular diagnostics, PCR, commercial kit, validation

## Abstract

In this report, we describe the first national scale multi-laboratory evaluation of monkeypox virus (MPXV) DNA commercial PCR kits. The objective of this study was to evaluate 2 kits by different diagnostic laboratories across Israel. Ten standardized samples were tested simultaneously using the Novaplex (15 laboratories) and Bio-Speedy (seven laboratories) kits. An in-house assay based on previously published reactions was used as reference. Comparison of the results showed high intra-assay agreement between laboratories, with small variations for most samples. The in-house assay had an analytical detection limit of less than 10 copies per reaction. While the 2 commercial kits were able to detect specimens with low viral loads similarly to the in-house assay, significant differences were observed, in the Cq values and relative fluorescence (RF), between the assays. The RF signal of the in-house and Bio-Speedy assays ranged between 5,000 and 10,000 RFU, while the signal in the Novaplex assay was less than 600 RFU. Due to the kit measurement protocol, the Cq values of the Bio-Speedy kit were 5 to 7.5 cycles lower than those of the in-house assay. On the contrary, the Cq values of the Novaplex kit were significantly higher than those of the in-house assay, with differences of 3 to 5 cycles per sample. Our results suggest that while all assays were similar in their overall sensitivity, direct comparison of Cq values between them may be misleading. To our knowledge, this is the first methodical evaluation of commercial MPX test kits. We therefore anticipate that this study would help diagnostic laboratories in choosing a specific MPX detection assay.

**IMPORTANCE** To the best of our knowledge, this study is the first methodical evaluation of commercial kits designed for Monkeypox virus detection. This was done by performing the same tests using the same sample set in multiple laboratories, simultaneously, on a national scale. It therefore provides important and unique information on the performance of such kits and provides a guideline for choosing the assay of choice for monkeypox virus diagnosis in a standard diagnostic laboratory. It also demonstrates potential complications when trying to compare the results of different assays, even when testing exactly the same samples, under identical conditions.

## INTRODUCTION

The recent monkeypox disease (MPOX) outbreak reached a worldwide scale within a few months, including Europe, North and South America, the Middle East, Australia, and large parts of Asia. This is in addition to Africa, where it has been endemic since its discovery in 1958 (1). Until October 2022, approximately 70,000 confirmed cases were reported, with actual numbers expected to be significantly higher (2). Due to its contagious nature on one hand, and the characteristic delayed onset of symptoms on the other hand, rapid detection of the virus is important for timely action of proper public health measures (3).

Diagnosis of MPOX was initially based on clinical and epidemiological criteria (4), and later, on several laboratory methods (3). The global smallpox vaccination campaign MPOX rendered serological diagnostics very challenging (5). Although serological MPOX-specific tests were reported (5), they could not be readily implemented in most of the relevant diagnostic laboratories.

Specific detection of monkeypox virus (MPXV), the causative agent of MPOX, using real-time PCR (rtPCR) is cheaper, faster, and can readily be adjusted for high throughput testing of very large numbers of samples, as was performed during the COVID19 pandemic (6). Furthermore, this approach can easily distinguish between different MPXV strains (7), which is currently not possible using serological tests. Lastly, rtPCR can detect the presence of viral DNA prior to the onset of symptoms, while serological reaction can only be detected 1 to 2 weeks after exposure. Taken together, these advantages render molecular testing the preferred choice for detecting MPXV infection. However, due to the fact that the disease was geographically limited until the present outbreak, availability of commercial MPXV detection kits was very limited, until recently.

MPOX was first reported in Israel during 2018, when an Israeli resident returned from Nigeria following exposure to infected rodents (8). This patient was isolated and recovered without any known subsequent infections. On 21^st^ of May 2022, the first MPOX-confirmed case was reported in Israel, and by October 2022, approximately 250 confirmed cases were reported (9). Thus far, laboratory MPOX diagnosis relied almost entirely on individual protocols. Following the COVID-19 pandemic, accelerated release of commercial PCR kits allowed standardized testing of viral diseases, including MPOX. However, thus far, no methodical evaluation of such commercial kits has been reported. In order to enable a country-wide diagnostic capacity, to respond to the increased circulation of MPXV, the Israel Ministry of Health initiated a national scale qualification procedure for MPX diagnosis, where participating laboratories performed a real-time PCR test on a standardized panel of samples. We describe here, for the first time, a national scale methodical evaluation of 2 such MPXV PCR commercial kits.

## RESULTS

### Evaluation of the CVL CDC-based in-house assay.

The sensitivity and the specificity of the “general” (GE) and clade 2 (formerly “West African” clade and referred to as WA hereafter) reactions were previously determined by Li et al. (2010) (7). Since we combined 3 reactions, we sought to ensure that the sensitivity of the multiplex assay was retained. Using a PCR product corresponding to the TNF-α receptor gene region (also designated MPX gp180 or J2R CDS), the assay sensitivity was evaluated. Serial dilutions of the PCR Standard control were tested in six repeats on different days. The analytic limit of detection that was accomplished in all repeats (100%), was 2 to 8 copies/μL for both MPXV reactions (Fig. S2).

The specificity of the reactions was previously established by Li et al. (2010) (7). In order to ensure the integrity of the multiplex assay specificity, samples of Varicella zoster virus (VZV and HHV3) and Measles virus (MeV), which can exhibit similar clinical presentation, and Orf virus, which is a Parapox virus. All samples were negative. This assay was then integrated into the diagnostic routine of the Israel Central Virology Laboratory (CVL) and is currently used to diagnose suspected MPXV samples. In the study described herein, this assay was used to set the reference values for the study with the samples panel. The standard sample panel was tested 6 times on different days, and the average (mean) values and standard deviation obtained are detailed in Table 1. As samples with MPXV Cq values greater than 31 contain diluted genetic material, their respective RNase P target (assay internal control) values were high, leading to a larger standard deviation (STDEV). For the MPXV reactions, the STDEV ranged between 0.36 to 1.36 cycles (Table 1).

**TABLE 1:** Average (Mean) and standard deviation Cq values of the standard MPXV samples panel obtained with the CVL assay based on the reactions described by Li et al. (2010) (7)

CVL assay			
Sample	GE (avg. Cq +/− S.D.)	WA (avg. Cq +/− S.D.)	RNAseP (avg. Cq +/− S.D.)
MPX-ST 1	21.9 ± 0.38	23.0 ± 0.32	28 ± 0.66
MPX-ST 2	25.7 ± 0.41	26.8 ± 0.36	32.9 ± 0.8
MPX-ST 3	27.4 ± 0.64	28.4 ± 04	33.6 ± 0.85
MPX-ST 4	29.6 ± 0.0.27	30.5 ± 0.38	36.1 ± 0.2
MPX-ST 5	29.4 ± 0.47	30.5 ± 0.35	36.2 ± 0.8
MPX-ST 6	32.1 ± 0.68	32.6 ± 0.74	38.2 ± 0.75
MPX-ST 7	34.9 ± 0.32	35.7 ± 0.27	37.3 ± 0.75
MPX-ST 8	35.4 ± 0.37	36.2 ± 0.4	34.5 ± 0.6
OrfV	N.D.^*a*^	N.D.	34.7
VZV	N.D.	N.D.	31.9 ± 0.21

a N.D., Not detected.

### Comparative evaluation of the Novaplex kit.

In order to evaluate the performance of the Seegene Novaplex MPX kit, the standardized MPXV samples panel was tested in parallel by 15 diagnostic laboratories across Israel. The list of participating laboratories is detailed in Table S3. During the kit evaluation trial, the panel samples were randomly labeled 1 to 10, without any indication of the expected result. For convenience, in this report, the samples were labeled according to their concentration. Sample MPX-ST-1 was the most concentrated and sample 8 was the most diluted.

Each laboratory performed the test in duplicate and the results were analyzed. All laboratories identified samples MPX-ST 1 to 7 as positive, and 8 identified marginal sample MPX-ST 8 as positive. The average Cq values of each sample, and the mean differences (delta) between the Cq value of the CVL assay and the Novaplex assay are shown in Table 2.

**TABLE 2:** Average Cq values and standard deviation of the samples panel tested with the Novaplex kit in 15 laboratories and the Bio-Speedy kit in 7 laboratories^*a*^

Novaplex assay		
Sample	Novaplex MPX (Avg. Cq +/− S.D.)	Cq_NVPLX_-Cq_CVL_
MPX-ST 1	26.5 ± 0.57	3.3
MPX-ST 2	29.8 ± 0.67	2.7
MPX-ST 3	32.8 ± 0.86	4
MPX-ST 4	35.4 ± 1.51	5.2
MPX-ST 5	34.3 ± 1.34	3.7
MPX-ST 6	37.5 ± 0.51	4.2
MPX-ST 7	40.4 ± 0.45	4.6
MPX-ST 8	41.3 ± 0.54	4.7
OrfV	N.D.^*b*^	N/A^*b*^
VZV	N.D.	N/A
Bio-Speedy assay		
Sample	Bio-Speedy MPX (Avg. Cq +/− S.D.)	Cq_BSP_-Cq_CVL_
MPX-ST 1	16.7 ± 0.35	−6.3
MPX-ST 2	20.1 ± 0.35	−6.6
MPX-ST 3	22.3 ± 0.4	−6.1
MPX-ST 4	24.6 ± 0.85	−5.9
MPX-ST 5	24.4 ± 0.35	−6.1
MPX-ST 6	27.5 ± 0.7	−5.1
MPX-ST 7	29.9 ± 0.5	−5.8
MPX-ST 8	28.8 ± 1.05	−7.4
OrfV	N.D.	N/A
VZV	N.D.	N/A

a In the right column, the Cq value differences between the average CVL in-house assay and the average Novaplex or Bio-Speedy assay are detailed.

b N.D., Not detected; N/A, Not applicable.

**FIG 1:**
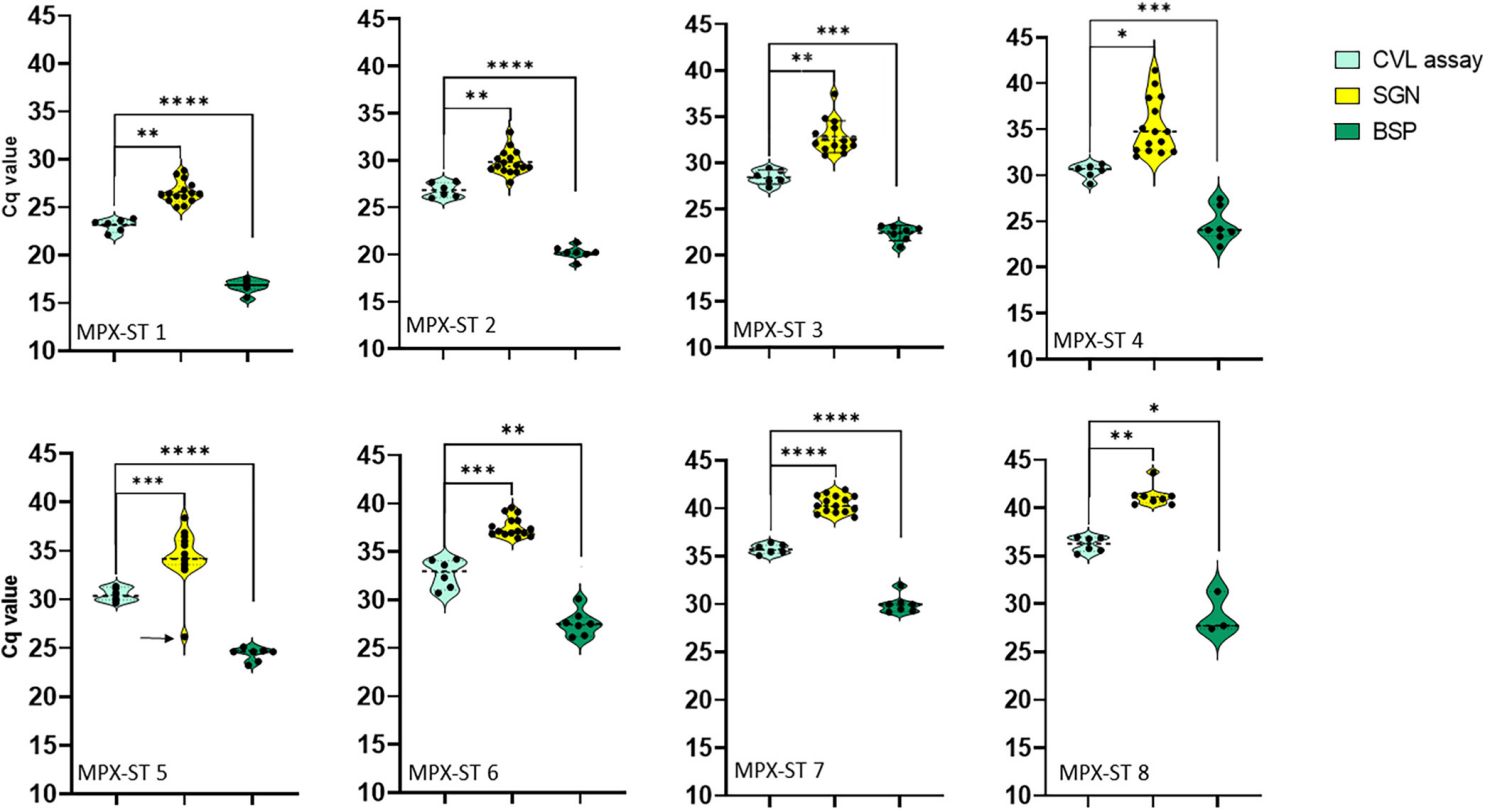
Comparison of the Cq values obtained with each assay, for each MPXV standard sample. Each individual value is represented by a single dot. The mean Cq in each plot is marked with a thick dotted line. The thin lines denote the quartile values of each sample. The distribution is depicted by the colored area for each assay, as indicated in the figure legend. SGN – Seegene Novaplex MPX kit, BSP – BioSpeedy MPX kit, CVL assay – the assay used in the Central Virology Laboratory, based on the MPXV reactions described by Li et al. (7). Statistical significance of the mean Cq value difference was calculated for each sample, between the CVL assay and each of the commercial kits. The *P*-value for each sample is indicated as follows: *, *P* < 0.05, **, *P* < 0.01, ***, *P* < 0.005, ****, *P* < 0.001. The *P*-value for sample 5 was calculated excluding the sample marked with an arrow (Cq = 26.2), which is 7–10 cycles lower than all other Novaplex values obtained for this sample.

The average and distribution of the Novaplex results, compared to the CVL assay and the Bio-Speedy (BSP) kit, are shown in Fig. 1. A detailed depiction of the results obtained by each laboratory is shown in Fig. S3. The values detailed under the “CVL In-house” test were obtained from the WA reaction, which is more stringent than the GE reaction (7). All laboratories correctly identified the VZV and Orf virus samples as negative for MPXV. Samples 1 to 7 were identified as positive by all laboratories (Fig. S3 and Table S4). Sample MPX-ST 8, which was similar to MPX-ST 7 in the CVL in-house assay, was identified as positive by 8 of the 15 laboratories. Within these 8 laboratories, in 3, it was identified in 1 of 2 repeats, as detailed in Table S4. The internal control of the kit was spiked into the samples and gave Cq values well below 45, indicating that the samples were not degraded and did not contain inhibitors.

Comparison of the Cq values obtained with the Novaplex kit from different laboratories, showed a varying degree of deviation (Table 2). Samples for MPX-ST 1,6,7,8 had a standard deviation of 0.9 to 1.1 cycles, while samples 2 to 5 had a S.D. of 1.15 to 3.02 cycles. The marginal sample MPX-ST 8 was identified as positive in 8 out of the 15 laboratories, with an average value of 41.3.

Notably, all results obtained by the Novaplex kit had significantly higher Cq values than those obtained with the CVL assay. The Cq differences of the average values obtained for each positive sample, ranged between 2.7 and 5.2 cycles (Fig. 1 and Table 2, right column). The statistical significance of the observed difference was confirmed by a paired *t* test, as detailed in Fig. 1.

### Comparative evaluation of the Bio-Speedy kit.

In order to evaluate the performance of the Bioeksen Bio-Speedy Monkeypox (MPXV) kit (https://www.bioeksen.com.tr/en/monkeypox-1), 7 laboratories tested the same sample panel with this kit. The sensitivity and uniformity of the kit performance was similar to that of the Novaplex kit, with all runs identifying 7 of the 8 positive samples, and sample MPX-ST 8 identified in 3 of the 7 laboratories (Fig. S4 and Table 2). The variation between the results ranged between 0.7 and 2.1 cycles (Table 2). The numerical values obtained by each laboratory are detailed in Table S5. Since the original samples used for the study were highly diluted, the RNase P control reaction was either very weak or negative for most samples and was therefore not analyzed here.

The Cq values obtained with the Bio-Speedy kit were consistently lower than those obtained with the in-house assay, due to the different test protocols (Fig. 1). The fluorescence reading starts on the first amplification cycle in the CVL in-house and Novaplex tests, while in the Bio-Speedy test, reading starts after 11 touchdown cycles (Fig. S1, Bio-speedy protocol). This resulted in an expected shift of 5.1 to 7.4 Cq values between the 2 assays (Fig. 1 and Table 2, right column). The statistical significance of the observed difference was confirmed by a paired *t* test, as detailed in Fig. 1.

### Comparison of the signal fluorescence between the different assays.

Comparison of the relative fluorescent signal generated by the different reactions showed marked differences between the Novaplex assay and the other 2 assays. Fig. S5 shows representative amplification curves and RFU values generated from 2 representative samples, MPX-ST 1 and 4. These samples gave RFU values of 6,000 to 10,000 units in the CVL and Bio-Speedy assays (Fig. S5A and B). The Novaplex assay results were analyzed twice: once directly with the CFX Maestro software (Bio-Rad), and once with the Seegene Viewer. This was done in order to compare the Cq values and fluorescence calculated by the 2 different tools. Importantly, both commercial kits recommend using the Bio-Rad CFX-96 cycler for their assays (www.bioeksen.com.tr, go.seegenetech.com/MPXV-assay). As detailed in the Methods section, the CVL assay was also run using this instrument. The analysis showed that both curves generated directly by the Bio-Rad CFX software and those generated by the Seegene Viewer software, gave RFU values of 200 to 550 units (Fig. S5C and D).

## DISCUSSION

Although monkeypox virus (MPXV) was discovered more than 60 years ago, and the first human case was diagnosed more than 50 years ago (4), molecular commercial detection kits were not widely available until the current worldwide outbreak. The MPOX outbreak in the USA during 2003 prompted the development of several molecular assays, using somewhat different technologies with varying specificities toward orthopox species and monkeypox in particular (10–14). In order to facilitate high throughput testing in a short time and affordable cost, and in the absence of an established information on the performance of such products, we set to establish a real-time PCR assay that can identify all MPXV strains, and indicated whether a positive sample belongs to Clade II (formerly WA clade) strain or not.

In order to perform a robust and informative assay, we combined the general (GE) and WA clade-specific (WA) reactions described by Li et al. (2010) (7) with an internal control reaction and demonstrated that the 2 MPXV reactions are sensitive and have very similar kinetics. A recent publication (15) suggested that changes in primers sequences developed by Li et al. (7) should be made, to render them more suitable to detect the currently circulating MPXV clade. In our reaction design, we addressed this by altering the sequence of the MPXV general (GE) reaction forward primer, already in May 2022, based on genomic analysis of currently circulating MPXV isolates. The WA reaction components showed perfect match to sequences of circulating MPXV samples and were therefore not altered. The assay’s sensitivity was consistent with that described by Li et al. (7), indicating that the combined multiplex retained the reactions’ sensitivity. Previous reports of real-time PCR assays reported similar sensitivity of 5 to 100 copies per reaction, with very high specificity (13, 14). Based on these reports, this assay was used as reference in this study. Upon the onset of the current MPOX outbreak, a rapid release of commercial kits was employed, to meet the urgent demand. To facilitate a national scale capability for MPOX diagnosis, 2 commercial assays that include an internal control reaction and can therefore be used as first-line test were evaluated.

Extensive experience gained during the COVID19 pandemic suggested that, though in some cases, different molecular kits showed very similar performance (16), several studies reported on significant differences between different test procedures (17–19). Our study focused on evaluating the performance of the 2 kits by simultaneous testing in different laboratories, but also on comparing the commercial kits and the CVL in-house assay. The comparison showed high reproducibility for most samples, with acceptable standard deviation values between laboratories, as shown in Fig. 1 and detailed in Table 2. Some of the weak samples resulted in higher S.D. values, as expected when the reproducibility of the assay declines. The uniformity of the results obtained by 15 laboratories (Novaplex kit) and 7 laboratories (Bio-Speedy kit) indicated that the products are sufficiently standardized, with a decline in reproducibility when testing marginal samples (Cq values higher than 40 in the Novaplex kit). The relatively high variability in the results of sample 4 (Fig. 1) is unaccounted for, since all samples originated from 1 pool of 5 clinical samples, inhibitory effects or other factors that affect the results should be similar in all samples. As the results of samples with similar or lower DNA concentrations were not as variable, this cannot be explained by differences in the kit quality. This variability was evident in the Novaplex kit and to a lesser extent in the Bio-Speedy kit.

Both assays correctly detected samples 1 to 7 in all repeats. Sample 8 was detected in 53% (8/15) of the Novaplex tests, and 43% (3/7) of the BioSpeedy tests. The numerical Cq values, however, were significantly different, as was previously reported for SARS-COV-2 detection kits (17). While the low values obtained with the Bio-Speedy kit resulted from the fluorescence measurement protocol, the values obtained with the Novaplex assay cannot be explained by the amplification protocol and are probably due to the proprietary Novaplex components and the reaction kinetics. These differences suggest that direct comparison between results of different assays cannot be performed and the “Cq shift” should be taken into consideration when analyzing results obtained with different assays. Importantly, all 3 assays correctly detected weak samples (marginal sample 8 was detected in some of the repeats), so under the examined conditions, the Cq differences did not affect the performance in terms of sensitivity.

In addition to the consistent shift in Cq values, the relative fluorescence (RFU values) varied significantly, between the in-house and Bio-Speedy assays, and the Novaplex assay, where the RFU values were between 10 and 20-fold lower. While the RFU value by itself does not usually affect the test result, a combination of low fluorescent signal and background “noise” can lead to misinterpretation of marginal results. The Novaplex kit accompanying software (Seegene Viewer for MPXV) is somewhat helpful in improving the test results but cannot completely overcome the potential problems associated with the low fluorescence. The results from the current comparison show that the apparent sensitivity of both kits was similar, with a slight advantage over the SGN kit: 7/15 compared with 3/7 for the BSP kit. However, our experience with similar SGN kits showed that the low RFU signal renders the analysis very challenging, in the absence of accompanying software (O. Erster, unpublished data). This contrasts with the in-house assay and the BSP kit, which can be analyzed directly using the CFX software and do not require additional post-reaction steps.

This study has several limitations related to the small number of tested samples (8 positive and 2 negative), and to the small number of repeats performed by each laboratory. Since the amount of standardized sample material and the availability of the kits were limited, multiple repeats that could determine the limit of detection more conclusively for each laboratory were not performed. However, since the results were uniform for most samples, and between labs, we believe that the data obtained herein are sufficiently significant to support the study’s conclusions. Another limitation is the small number of clinical samples that was used; in order to standardize the evaluation, a pool of 5 relatively concentrated samples was needed as a starting point, and the availability of such samples was limited at the time of the study. This somewhat limits the ability to assess the robustness of the assays, as different samples may contain different compositions of inhibitors that can affect the test results.

In conclusion, both kits can be used for laboratory diagnosis of MPXV, providing that they will be used according to the manufacturer’s instructions. However, the weak fluorescent signal and delayed Cq values obtained using the Novaplex kit require consideration when testing suspected samples that give marginally positive results. Additionally, comparison of the results obtained by different assays require proper adjustments that consider the different measurement protocols. It is expected that if the MPOX outbreak persists, existing products will be improved and additional products will become available, thereby improving the diagnostic capacity of monkeypox infections. It is therefore advisable for diagnostic laboratories to consider the aspects addressed herein, when choosing which assay to use for MPXV detection.

To the best of our knowledge, this is the first national scale, comparative evaluation of commercial MPXV Real-time PCR detection kits.

## MATERIALS AND METHODS

### Sample origin and preparation.

The samples used for the study were prepared from the remaining DNA extractions of monkeypox virus clinical samples that were obtained from patients during routine diagnostics. Patients were examined in the Sheba Medical Center as suspected monkeypox disease (MPOX) patients. Samples were used for this study under the institutional committee of the “Sheba ethical board” under protocol number 9481-22-SMC. The samples were collected directly from skin lesions using standard collection swabs, which were immediately placed in universal transport medium (UTM) tubes (https://www.copanusa.com/). Once collected, samples were transferred to the laboratory and incubated at 4°C until processing.

DNA extraction was performed using the PSS MagLEAD nucleic acid purification system (https://www.pss.co.jp/english/product/magtration/lead6-12gc.html). Five individual samples were pooled together, and the pool was diluted using IDTE buffer pH 8 (IDT, https://eu.idtdna.com/) to generate a set (panel) of 8 standard samples in different viral DNA concentrations. The initial sample pool gave a Cq value of approximately 20 using the CVL in-house assay. According to the calculated standard control calibration, the initial concentration of the pooled samples stock (Cq = 20), was approximately 1.8 × 10^5^ copies/μL. The dilutions of the panel from the original pool sample are detailed in Table S1.

Additional 2 samples of varicella zoster virus (VZV) and orf virus (OrfV) were included in the sample set as monkeypox-negative samples. Each pool dilution was aliquoted and stored in −80°C.

### CVL MPXV qPCR assay.

The reference test that was used in this study was based on the reactions developed by Li et al. (2010) (7) for general detection of MPXV and specific detection of Clade II (formerly designated “West Africa”) MPXV strain. The primers and probes described by Li et al. (7) were incorporated into a single assay, together with primers and probe targeting human RNAseP as an internal control (https://www.cdc.gov/coronavirus/2019-ncov/lab/rt-pcr-panel-primer-probes.html). The forward primer of the GE reaction was modified from the original design, so that it contained either A or G in position 6 from the 5’p, to complement recently sequenced samples. The sequence of this primer was therefore 5′-GGAAA**R**TGTAAAGACAACGAATAC-3′, where R (A or G) replaces the original A. The multiplex assay was tested for its sensitivity and specificity, using serial dilutions of a clinical sample, and clinical non-MPXV samples of other pathogens. Table S2 details the components that were assembled in the reaction mix.

The following run protocol was used on Bio-Rad CFX-96 thermal cycler:

(i) 45°C for 5 min., (ii) 95°C for 4 min., 40 X [(iii) 95°C for 4 min., (iv) 58°C for 10 sec., (v) 60°C for 0:10+ Plate Read]. Graphical representation of the protocol is shown on Fig. S1.

The fluorescence threshold was set at 200 RFU, which is the default threshold we use for diagnostic assays.

### Standard control.

In order to quantify the real-time PCR results to a number of copies in a tested sample, we generated a standard control for the assay. A 1050 bp product corresponding to the J2R gene was cloned from a positive clinical sample, using primers MPXV TNF-α R Fwd (5′-CATGAGGTCCGTATTATACTCG-3′) and TNF- α R Rev (5′-CTTACTATAAGTGGGTGGGATTC-3′). The primer binding positions were 194,072 (Fwd) and 195,122 (Rev) in sequence no. NC_003310. The product was purified and quantified using Nanodrop spectrophotometer (Thermo Fisher). The number of target copies in one nanogram of PCR product was calculated using the online tool at http://www.scienceprimer.com/copy-number-calculator-for-realtime-pcr and was determined to be 8.69 × 10^8^ copies. The stock product was then diluted to a concentration of 5 × 10^6^ copies/μL, and the diluent was used for creating the dilutions used for the kits evaluation. The diluted standard was aliquoted and stored at −20°C.

### Seegene Novaplex MPX kit.

The Seegene Novaplex kit Cat. No. R-PX10325Z for detection of MPXV DNA (https://www.seegene.com/assays/novaplex_mpxv_assay) was used according to the manufacturer's instructions. Importantly, the kit internal control was spiked into the samples before setting the reaction. Graphical representation of the amplification program used on Bio-Rad CFX-96 thermal cycler is shown on Fig. S1. The Novaplex assay was analyzed either directly using the CFX-96 Maestro software (www.bio-rad.com) or using the Seegene MPX Viewer software (www.seegene.com/software/).

### Bio-Speedy Monkeypox kit.

The Bio-Speedy Monkeypox virus qPCR kit (Bioeksne.com.tr, Cat. No. BS-MPV-25) was prepared and used according to the manufacturer's instructions (bioeksen.com.tr/en/Monkeypox-1). Graphical representation of the amplification program used on Bio-Rad CFX-96 thermal cycler is shown on Fig. S1. The Bio-Speedy (BSP) assay results were analyzed according to the manufacturer’s instructions using the CFX-96 Maestro software.

### Statistical analysis.

For each set of repeats for a specific sample tested using the same assay, the mean Cq value, and standard deviation were calculated. Comparisons between the results of each assay were performed using the GraphPad Prism software (www.graphpad.com/scientific-software/prism/). Analysis of the difference between the Cq values of each assay for each sample was performed using a paired *t* test.
