# Supplementary material for: A Multi-Laboratory Evaluation of Commercial Monkeypox Virus Molecular Tests
Source: Microbiol Spectr. 2023 May 4;11(3):e00225-23. doi: 10.1128/spectrum.00225-23 (PMC10269769; doi:10.1128/spectrum.00225-23)
Supplement: Supplemental file 1 — Tables S1 to S5 and Fig. S1 to S5. Download spectrum.00225-23-s0001.pdf, PDF file, 1.2 MB [file spectrum.00225-23-s0001.pdf]

SUPPLEMENTAL MATERIAL

A Multi-Laboratory Evaluation of Commercial Monkeypox Molecular Tests

Oran Erster[1§], Itzchak Levy[2], Areej Kabat[1], Batya Mannasse[1], Virginia Levy[1], Hadar Assraf[1], Roberto Azar[1], Haim Ben-Zvi[3], Rita Bradenstein[4], Olga Bunder[5] , Ayman Fadeela[6], Ayelet Keren-Naus[7], Avi Peretz[8,19], Diana Roif-Kaminsky[9], Lolu Saleh[3], Licita Schreiber[10], Orna Schwartz[11], Pninit Shaked-Mishan[12], Nadav Sorek[13], Merav Strauss[14], Rachel Steinberg[7], Orit Treygerman[15], Simona Zisman-Rozen[16] ,Ruth Yishai[17], Noa Tejman-Yarden[17], Ella Mendelson[1,18], Danit Sofer[1]

[1]Central Virology Laboratory, Public Health Services, Ministry of Health, Chaim Sheba Medical Center, Ramat Gan, Israel
[2]Infectious Diseases Unit, Chaim Sheba Medical Center, Ramat Gan, Israel
[3]Beilinson-Rabin Medical Center, Petach Tikva, Israel
[4]Kaplan Medical Center, Rehovot, Israel
[5]Shamir Medical Center, Beer Yaacov Zerifin, Israel
[6]Meir Medical Center, Kfar Sava, Israel
[7]Soroka Medical Center, Be'er Sheva, Israel
[8]The Baruch Padeh Medical Center, Poriya, Tiberias, Israel
[9]Barzilai Medical Center, Ashkelon, Israel
[10]Maccabi HealthCare Central Laboratory, Rehovot, Israel
[11]Wolfson Medical Center, Holon, Israel
[12]Carmel Medical Center, Haifa, Israel
[13]Assuta Ashdod University hospital, Ashdod, Israel
[14]Emek Medical Center, Afula, Israel
[15]Me'uchedet HealthCare Central Laboratory, Lod, Israel
[16] Galilee Medical Center, Nahariya, Israel
[17]Department of Laboratories, Public Health Services, Ministry of Health, Jerusalem, Israel.
[18]School of Public Health, Sackler Faculty of Medicine, Tel-Aviv University, Tel-Aviv, Israel.
[19]The Azrieli Faculty of Medicine, Bar-Ilan University, Safed, Israel

[§]Corresponding author contact information: oran.erster@sheba.health.gov.il

Sample	Calculated Dilution
Pool stock	1.0E+00
MPX-ST 1	1.0E+01
MPX-ST 2	1.0E+02
MPX-ST 3	5.0E+02
MPX-ST 4	1.5E+03
MPX-ST 5	1.5E+03
MPX-ST 6	5.0E+03
MPX-ST 7	5.0E+04
MPX-ST 8	7.5E+04

Supplementary Table S1. Dilutions of pooled MPX standard samples**.**

**Component**	**Volume per reaction (µl)**	**Final concentration**
2X SensiFast DNA mix	10	
H_2O	2.7	
G2R G Fwd 40µM	0.3	0.6µM
G2R G Rev 40µM	0.3	0.6µM
G2R G probe 20µM FAM	0.3	0.3µM
G2R WA Fwd 40µM	0.3	0.6µM
G2R WA Rev 40µM	0.3	0.6µM
G2R WA probe 20µM HEX	0.3	0.3µM
RNASE P-F 40µM	0.15	0.15µM
RNASE P-R 40µM	0.15	0.15µM
RNASE P-P 20µM Cy5	0.2	0.2µM
Total master mix volume	15	
DNA	5	
**Total reaction volume**	**20**	

Supplementary Table S2. Components of the multiplex CVL MPX assay.

**Laboratory institutional affiliation**	**Lab abbreviation**
Assuta Ashdod University hospital	Assuta
Barzilai Medical Center	Barzilai
Beilinson-Rabin Medical Center	Beilinson
Carmel Medical Center	Carmel
Israel Central Virology Laboratory	CVL
Galilee Medical Center	Galilee
Emek Medical Center in Afula	Haemek
Kaplan Medical Center	Kaplan
Maccabi Helathcare Central Laboratory	Maccabi
Meir Medical Center	Meir
United ("Me'uhedet") Helathcare Central Laboratory	Me'uhedet
Baruch Padeh Medical Center AKA Poriya Medical Center	Poriya
Shamir Medical Center, formerly Assaf Harofeh Medical Center	Shamir
Soroka Medical Center	Soroka
Wolfson Medical Center	Wolfson

Supplementary Table S3. List of participating laboratories and their institutional affiliation.

Test	Lab\Sample	1	2	3	4	5	6	7	8	OrfV	VZV
**CVL assay**	CVL In-house	23.2	27.1	28.9	30.2	30.6	33.2	35.8	36.6	N.D.	N.D.
**Novaplex**	Assuta	28.4	31.6	34.5	37	36.5	39.1	41.9		N.D.	N.D.
**Novaplex**	Barzilai	26.3	29.7	31.7	33.5	36	36.8	40.2		N.D.	N.D.
**Novaplex**	Beilinson	26	29.3	32.6	32.8	34.2	37	40.7	40.4	N.D.	N.D.
**Novaplex**	Carmel	25	28.7	30.8	32.7	34.1	38.1	39.3	41	N.D.	N.D.
**Novaplex**	CVL	26.3	30.1	32.4	33.7	34.1	36.8	40.2	41.3	N.D.	N.D.
**Novaplex**	Emek	28.8	33	34.8	41.5	26.2	39.4	41.3	43.8	N.D.	N.D.
**Novaplex**	Galilee	26.2	30.2	32.1	40	34.7	36.7	41.3		N.D.	N.D.
**Novaplex**	Kaplan	26.1	28.9	37.5	38.5	33.5	37.2	41.2	41.4	N.D.	N.D.
**Novaplex**	Maccabi	26.4	28.7	31.9	38.6	36.9	38.1	39.9	40.8	N.D.	N.D.
**Novaplex**	Meir	25.6	29	31	32.5	33.1	36.7	39.6		N.D.	N.D.
**Novaplex**	Me'uhedet	28	30.7	33.2	34.8	38.4	37.5	41.6		N.D.	N.D.
**Novaplex**	Poriya	27.2	30.8	33.8	34.8	35.6	39	40.8	41.3	N.D.	N.D.
**Novaplex**	Shamir	24.9	27.6	32.7	32.1	33.2	37	39.7		N.D.	N.D.
**Novaplex**	Soroka	26.7	29.4	31.9	35.2	34.2	36.5	39.5	40.4	N.D.	N.D.
**Novaplex**	Wolfson	25.6	29.2	31.5	32.6	33.7	36.3	39		N.D.	N.D.

Supplementary Table S4. Novaplex comparative test. Average Cq values obtained from 15 diagnostic laboratories for each panel sample using the Novaplex (SGN) kit. All laboratories tested the same samples using the same protocol. N.D. – Not detected

Test	Lab\Sample	1	2	3	4	5	6	7	8	OrfV	VZV
**CVL In-house**	**CVL In-house**	21.9	25.7	27.4	29.6	29.4	32.1	34.9	35.4	N.D.	N.D.
**BSP**	**Beilinson**	17.3	21.2	22.6	24.1	24.7	27.2	30.1		N.D.	N.D.
**BSP**	**CVL**	15.4	18.9	20.8	22.3	23.3	26	29.2	27.4	N.D.	N.D.
**BSP**	**Emek**	16.6	20.5	22.2	27.5	23.7	26.2	31.9		N.D.	N.D.
**BSP**	**Carmel**	16.4	20.1	21.7	23.4	24.8	30	29.4	27.7	N.D.	N.D.
**BSP**	**Shamir**	17.1	19.9	23.1	23.9	24.7	27.4	29.1		N.D.	N.D.
**BSP**	**Soroka**	17.4	20.1	22.8	24.2	25.2	27.5	29.9	31.3	N.D.	N.D.
**BSP**	**Wolfson**	16.8	20.1	23	26.8	24.7	28.2	29.9		N.D.	N.D.

Supplementary Table S5. Bio-speedy comparative test. Average Cq values obtained from seven diagnostic laboratories for each panel sample using the Bio-speedy (BSP) kit. All laboratories tested the same samples using the same protocol. N.D. – Not detected

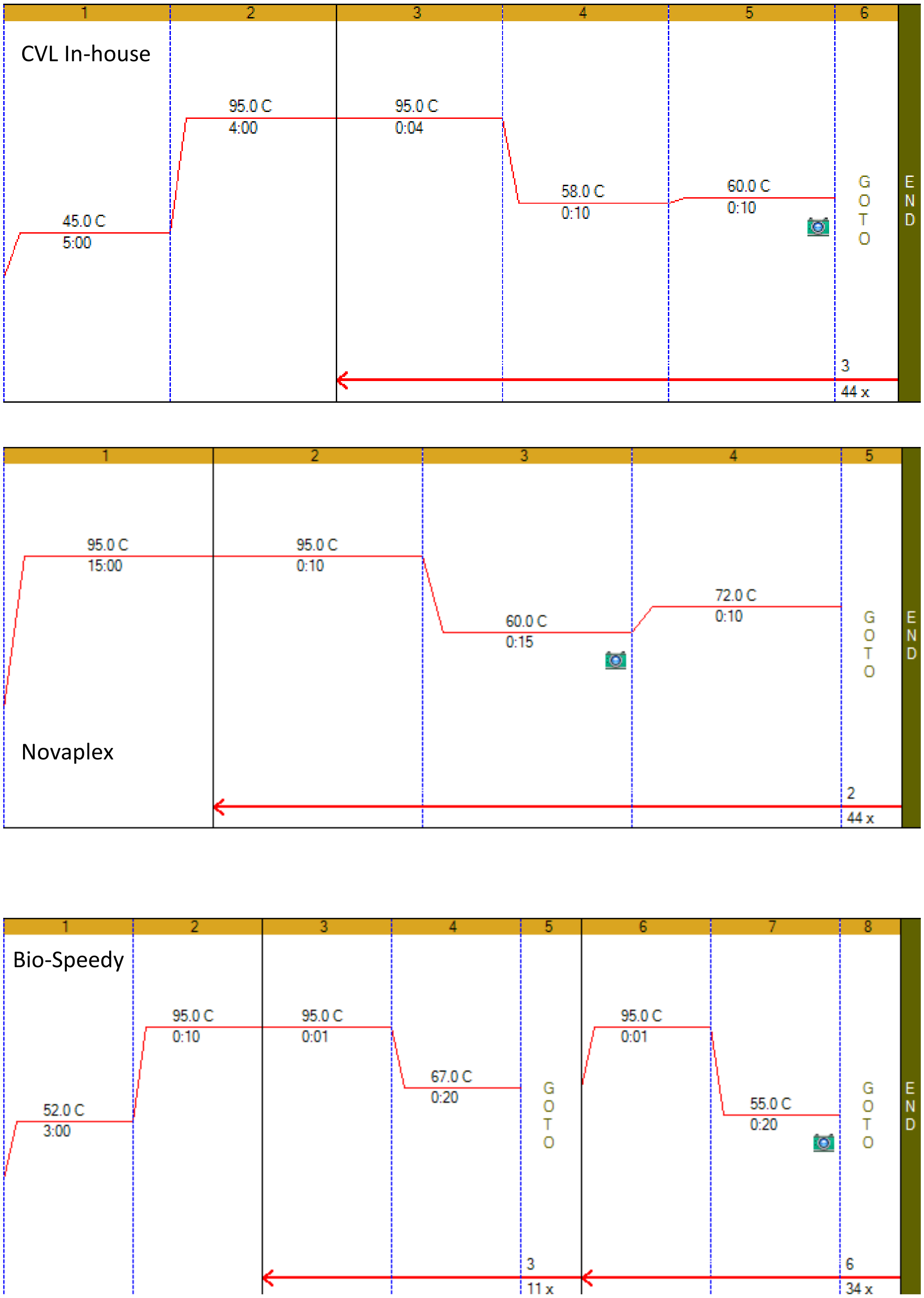

<u>Supplementary Figure S1.</u> Graphical representation of the cycling protocol of each assay. While fluorescent recording starts on the first amplification cycle in the CVL and Novaplex protocols (top and central panels), in the Bio-Speedy protocol (bottom panel) it starts after 11 cycles.

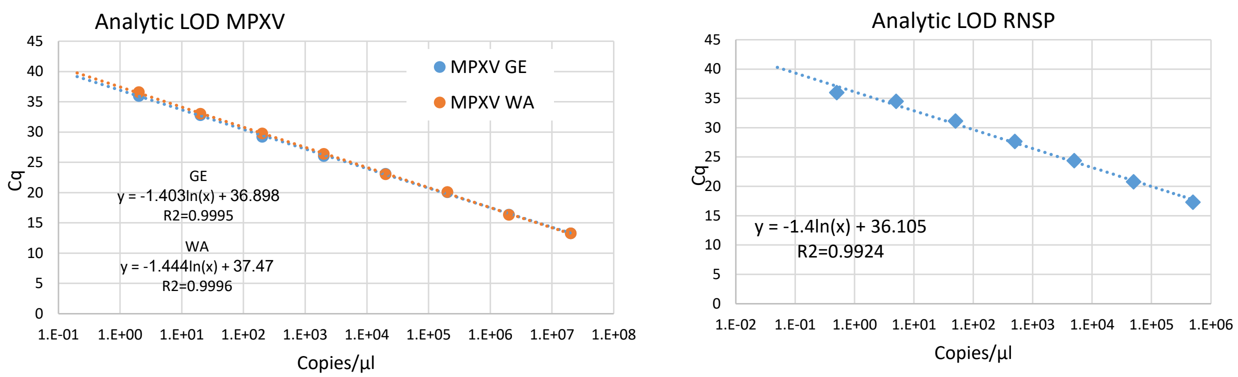

<u>Supplementary Figure S2.</u> Analytic limit of detection (LOD) curves of standard quantified controls. Curves were plotted for the MPX GE and WA reactions (A) and for the human RNAse P reaction that was used as an endogenous control. Serial dilutions of quantified control DNA were tested in triplicates and the Cq values were plotted against the calculated concentration. The formula of the derived regression line and the R^2 value of the curve are shown for each reaction.

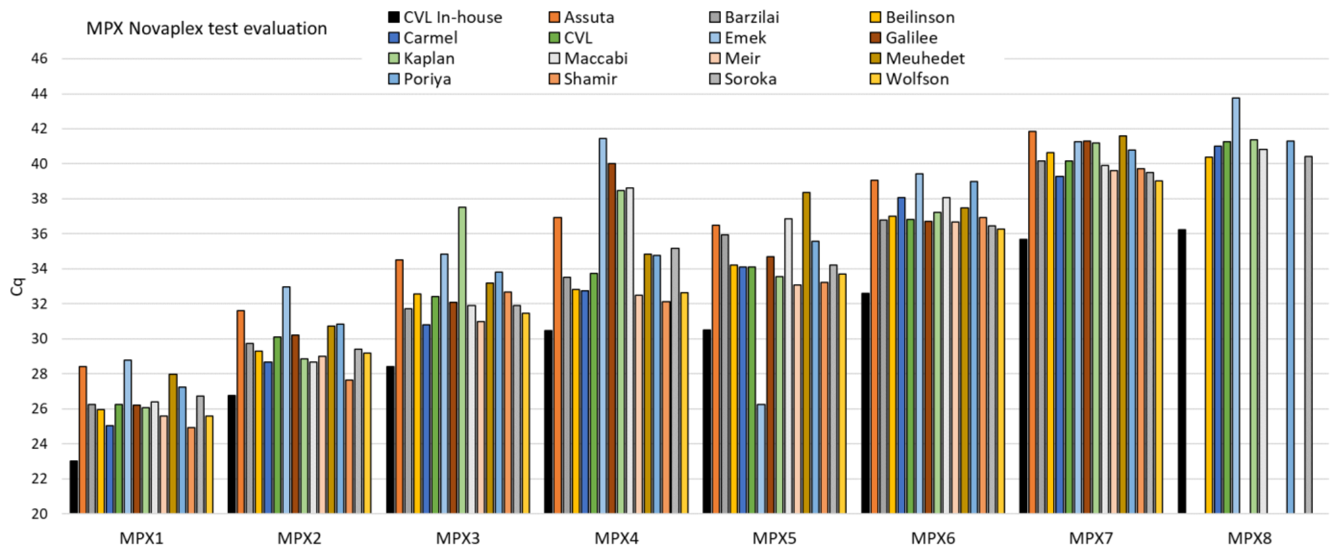

<u>Supplementary Figure S3.</u> Average Cq values obtained from each participating laboratory, of each MPX panel sample using the Seegene Novaplex kit. The average value represents at least two separate repeats performed in each laboratory.

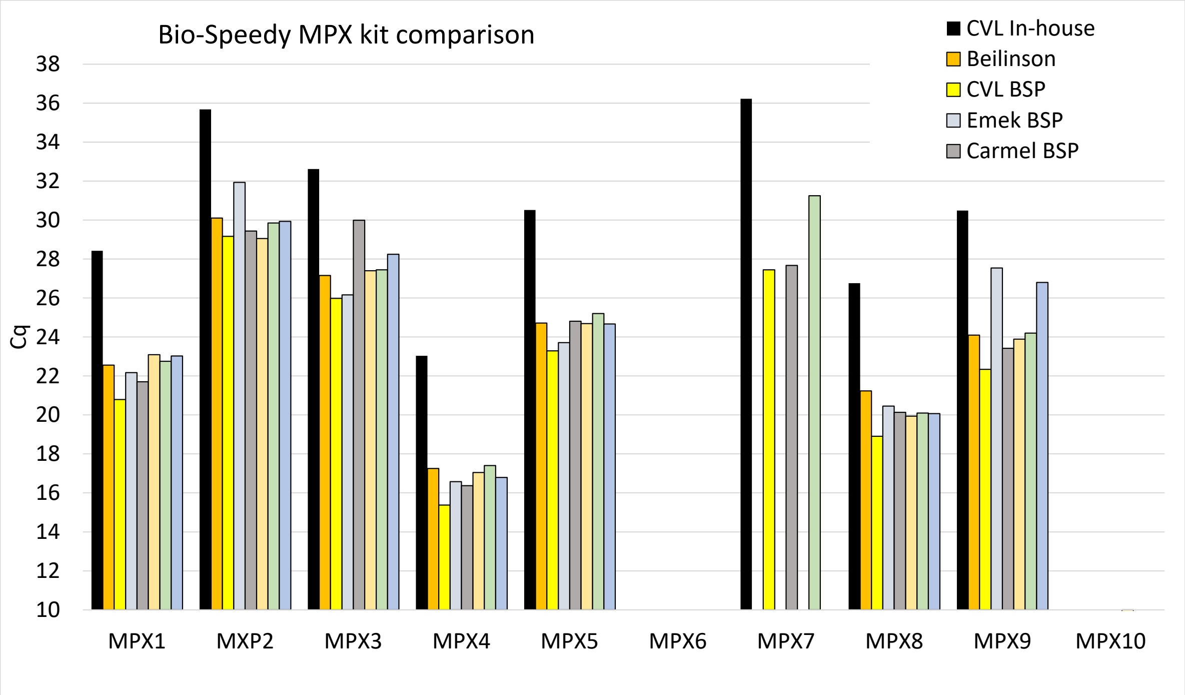

Supplementary Figure S4. Average Cq values obtained from each participating laboratory, of each MPX panel sample using the Bioeksen Bio-Speedy (BSP) kit. The average value represents at least two separate repeats performed in each laboratory.

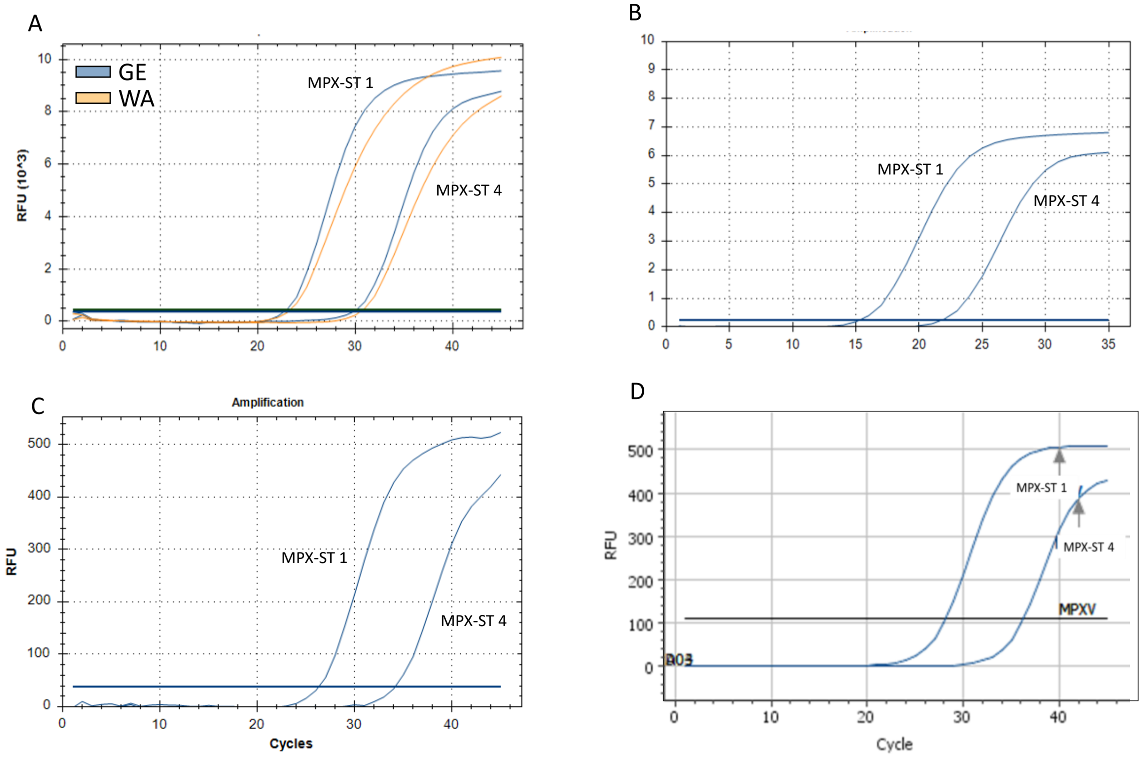

Supplementary Figure S5. Amplification curves of the CVL, Bio-Speedy and Novaplex assays. Duplicate curves of representative panel samples MPX4 and MPX9 are shown, as obtained with the CVL In-house assay (A), the Bio-Speedy assay (B), the Novaplex raw analysis (C), and the Novaplex accompanying software (Seegene viewer) analysis (D). RFU – Relative fluorescence units.
